# Estimate of Novel Influenza A/H1N1 cases in Mexico at the early stage of the pandemic with a spatially structured epidemic model

**DOI:** 10.1371/currents.RRN1129

**Published:** 2009-11-11

**Authors:** Vittoria Colizza, Alessandro Vespignani, Nicola Perra, Chiara Poletto, Bruno Gonçalves, Hao Hu, Duygu Balcan, Daniela Paolotti, Wouter Van den Broeck, Michele Tizzoni, Paolo Bajardi, Jose J. Ramasco

**Affiliations:** ^*^ISI Foundation, Turin, Italy; ^†^Indiana University; ^‡^Complex Networks and Systems Research School of Informatics and Computing; ^§^Institute for Scientific Interchange Foundation, Turin; ^¶^Center for Complex Networks Systems Research; ^#^Department of Physics and Center for Complex Networks and Systems Research, Indiana University; ^**^Indiana University, Bloomington, IN; ^††^Institute for Scientific Interchange - ISI; ^‡‡^ISI Foundation; ^§§^Computational Epidemiology Laboratory, ISI Foundation, Turin, Italy; ^¶¶^Computational Epidemiology Laboratory, Institute for Scientific Interchange and ^##^Complex Networks Lagrange Laboratory and Computational Epidemiology Laboratory, ISI Foundation, Turin, Italy

## Abstract

Determining the number of cases in an epidemic is fundamental to properly evaluate several disease features of high relevance for public health policies such as mortality, morbidity or hospitalization rates. Surveillance efforts are however incomplete especially at the early stage of an outbreak due to the ongoing learning process about the disease characteristics. An example of this is represented by the number of H1N1 influenza cases in Mexico during the first months of the current pandemic. Several estimates using backtrack calculation based on imported cases from Mexico in other countries point out that the actual number of cases was likely orders of magnitude larger than the number of confirmed cases.

Realistic computational models fed with the best available estimates of the basic disease parameters can provide an ab-initio calculation of the number of cases in Mexico as other countries. Here we use the Global Epidemic and Mobility (GLEaM) model to obtain estimates of the size of the epidemic in Mexico as well as of imported cases at the end of April and beginning of May. We find that the reference range for the number of cases in Mexico on April 30th is 121,000 to 1,394,000 in good agreement with the recent estimates by Lipsitch et al. [M. Lipsitch, PloS One 4:e6895 (2009)]. The number of imported cases from Mexico in several countries is found to be in good agreement with the surveillance data.

## Introduction

An unprecedented global effort in surveillance has been carried out by national and international health agencies for the current novel Influenza A (H1N1) pandemic [1]. Along with the institutional efforts, a combination of media attention and new mechanisms for information retrieval, such as Internet, has allowed the prompt gathering of large amount of data that allow for the first time the real-time analysis of a pandemic in such a detailed way. Unfortunately, the access and availability of data does not imply reliability or accuracy. A paramount example is provided by the time evolution of the number of cases in each country. In the case of influenza, the presence of asymptomatic cases or of cases showing mild symptoms who might not seek for medical attention lead to an underascertainment of cases that is hard to estimate. Moreover, the monitoring of cases is expected to change with time, from an enhanced surveillance at the start of the epidemic, followed by the ascertainment of more severe cases, needing medical attention, hospitalization, and also confirmation of the infection. This is due to the large increase in the number of cases overwhelming surveillance systems, which therefore relax their requirements as the epidemic progresses due to limited capacity and high costs associated with systematic serological testing. For these reasons, after the very initial stage of the outbreak, the number of confirmed cases tends to be a gross underestimation of the actual number of infections [2] [3] [4] [5]. On the other hand, reliable figures for the actual number of cases is the key to the estimate of parameters such as the mortality, morbidity or hospitalization rates that are on their turn crucial in the policy making process.  A paramount example of this issue is provided by the worries caused by the early estimate of the fatality rate of the current H1N1 pandemic from the Mexican data. As it turned out later, this number was inflated because the confirmed cases of infections were grossly underestimated in Mexico [2]. In particular, two studies have assessed the size of the epidemic in Mexico by analyzing the number of H1N1 infected travelers arriving from Mexico detected by the surveillance systems of other countries in their attempt to contain the epidemic [5] [6].  Both papers find that the estimate for the number of cases in Mexico at the end of April early May is orders of magnitude larger than those confirmed by Mexican authorities. In particular, the calculation of Ref. [6] that uses the most updated data source gives a lower bound for the number of cases that is between 113,000 and 375,000 cases, to be compared with the official report that indicate 3,350 confirmed cases [7].

Here, we use the Global Epidemic and Mobility (GLEaM) model [8] [9] to provide a computational ab-initio evaluation of the early size of the outbreak in Mexico and the ﬂuxes of infected travelers to other countries. The GLEaM model is a spatially structured metapopulation epidemic model [8] [9] [10] [11] [12] [13] [14] [15] [16] [17] [18] [19] [20] [21], that allows the generation of stochastic realizations of the worldwide unfolding of the epidemic, with mobility processes entirely based on real data. Once the disease parameters and initial conditions based on available data are defined, the model generates in-silico epidemics for which we can gather information such as prevalence, morbidity, number of secondary cases, number of imported cases and many others for each subpopulation and with a time resolution of one day. In Ref. [9], the GLEaM model has been used to perform a Maximum Likelihood Estimate (MLE) of the transmission potential of the current H1N1 pandemic. Here we use the best estimate parameters from Ref.[9] to simulate 2x10^3^ stochastic simulations of the current pandemic and provide an estimate of the number of H1N1 cases in Mexico at the date of May the 8th. This is an ab-initio computational estimate and to further cross-validate our results, we compare the number of infected individuals traveling from Mexico to other countries from our simulations and compare these numbers with surveillance reports [6] [22] [23] [24]. We find in our simulations that in Mexico as of the date of May the 8th the symptomatic cases 95% reference range is 121,000 to 1,394,000. This value is in good agreement with the lower bound estimate of Ref. [6]. The number of infected individuals with travel history from Mexico in countries such as US, Canada, Spain and UK is also within the confidence range of our simulations. These results provide further support to the claim that the number of confirmed H1N1 cases in Mexico is only a very small fraction of the actually occurred cases. 

## Materials and Methods

The global epidemic and mobility metapopulation (GLEaM) model is based on a geographically structured metapopulation approach [8] [9] [10] [11] [12] [13] [14] [15] [16] [17] [18] [19] [20] [21] [25]. GLEaM is composed of three layers. The first one is the population layer that integrates distinct census areas for a total of 3362 subpopulations in 220 countries of the world. The census areas are defined by a Voronoi-like tessellation process that partition the world surface. Given a set of points S in the plane, which are the Voronoi sites, each site s has a Voronoi cell V(s) consisting of all points closer to s than to any other site. In our partition we use 3362 major transportation hubs in the world as the Voronoi sites. The boundaries of each subpopulation are defined by all the points in the plane that are equidistant to two sites. For each area defined by the cell boundaries and the Voronoi process, the population is obtained from the site of the ”Gridded Population of the World” project of SEDAC (Columbia University)[26] that provides population estimates worldwide for cells of 15 × 15 minutes of arc. The second layer of the model is composed by the human mobility flows among the census areas. We consider both commuting flows collected from various sources in more than 30 countries and the airline traffic provided by IATA [27] and OAG [28]. Further details concerning the composition of GLEaM and the integration of its three layers for a practical simulation of epidemic spreading are given in Ref. [9]. The third layer of the model concerns the disease dynamics used to model the disease evolution. We adopt a SEIR-like compartmentalization framework in which separate compartments for symptomatic traveling and not traveling, as well as asymptomatic individuals are included in each different subpopulations. The infection dynamics takes place within each subpopulation and assumes the classic influenza-like-illness compartmentalization in which each individual is classified by one of the discrete states such as susceptible, latent, infectious symptomatic, infectious non-symptomatic or permanently recovered/removed.  All transitions are modeled through binomial and multinomial processes to ensure the discrete and stochastic nature of the processes. A full definition of the model is reported in Ref [9]. 

It is also worth stressing here some of the model assumptions. The model is not an agent-based model and does not include additional structure within a subpopulation, therefore it cannot provide detailed information at the level of households or workplaces. The fraction of infected population is likely overestimated because of the assumptions of an entirely susceptible populations and of subpopulations with homogeneous mixing. Current data on the severity of the pandemic has revealed an age pattern for influenza attack rate shifted towards the younger age classes of the population [5] [29] [30] [31] [32] [33] [34], suggesting a possible presence of cross-immunity between the H1N1 pandemic strain and preexisting influenza viruses in the elderly [35] [36] [37], besides other mechanisms. This possibility is however still under exploration and no reliable estimates are available, therefore we assumed a fully susceptible population to study the initial stage of the outbreak, following previous studies on H1N1 pandemic [5] [6] [38] [39].   

The spreading rate of the epidemic is governed by the basic reproduction number and the generation interval of the specific viral strain considered. In order to obtain best estimate for these parameters the model has been used to perform a MLE of the parameters against the actual chronology of newly infected countries [9]. This methodology considers a Monte Carlo generation of the distribution of arrival time of the infection in each country based on the analysis of one million worldwide simulations of the pandemic evolution with the GLEaM model. This analysis provides the maximum likelihood estimates for the basic parameters of the H1N1 such as the reproductive number R_0_ and the basic model parameters ε and μ defining the inverse average exposed and infectious time durations, respectively. In the following we consider as the baseline case the set of parameters defined by the best estimates: ε^ -1^=1.1 days, μ ^-1^=2.5 days, R_0_=1.75 [9], consistent with the estimates of Ref. [38]. Asymptomatic individuals are assumed to represent 1/3 of the total cases, and have a reduced transmissibility of ½ with respect to symptomatic individuals [9] [38] [40] [41]. The sensitivity analysis and confidence interval for those values are reported in Ref.[9]. For this set of parameters the model generates quantities of interest such as the profile of the epidemic behavior in each subpopulation or the number of imported cases. In the following, simulation results are aggregated at the level of the country for a direct comparison with the empirical data available. The initial conditions of the epidemic are defined by setting the onset of the outbreak near La Gloria in Mexico on February 18^th^, 2009, as reported by official sources [36] and analogously to other works [8]. We tested different localization of the first cases in census areas close to La Gloria without observing relevant variations with respect to the observed results. In Mexico we also consider the control measures implemented in the country starting April 24^th^ and ending May 10th following Ref. [2], as those might affect the spreading to other countries [9]. Here we focus on the cumulative number of cases observed in Mexico at the date of April the 30^th^ and the imported cases in the UK, US, Brazil, Germany and France. Each simulation represents a stochastic realization of the process and we aggregate data on 2x10^3^ realizations providing reference ranges for all quantities. The data of imported cases are compared with those reported in Ref. [6] for the UK and the US. The data for France was obtained from Ref. [23], those for Germany from Ref. [22] and those for Brazil from the reports at the site of the Health Department of the Brazilian Government [24]. 

## Results and Discussion

By using GLEaM it is possible to provide a model estimate of the number of imported cases arriving from Mexico to a set of selected countries. The estimated 99% reference range is shown in Table 1. The dates and target countries are chosen to facilitate the comparison with the numbers found in the literature [6] [22] [23] [24]. The numbers shown in the Table refer to the importantion of infected/exposed individual traveling from Mexico in one of the listed countries as of the date of May the 8^th^.  Only 2/3 of  the exposed travelers are then considered in the cumulative number of cases as only this fraction will eventually develop symptoms, according to the model assumptions. The numbers of imported cases to each country are typically small, and as such prone to large stochastic ﬂuctuations. However the surveillance values are all within the 99% reference ranges of the 2,000 realizations of our model. We will provide elsewhere a full sensitivity analysis of the results but we observe very small variations with respect to the presented results in the range of parameters explored. This is because any MLE for R_0_ and generation interval tend to optimize the growth rate with respect to the epidemic timeline thus producing very similar results in the early spreading of the epidemic. We have also considered that in the US the travel history is known only for 50% of the confirmed cases. The simple extrapolation that provides a twofold estimate of imported cases (in brackets in Table 1) is however still compatible with the reference range of our stochastic simulations. 

Table 2 shows GLEaM predictions for the size of the epidemic in Mexico on April 30^th^ and compare the results with the estimations of Refs. [5] and [6]. We provide the 95% reference range over 2,000 realizations. The obtained range includes the lower bound estimate of Ref [6]. Our median value for the number of asymptomatic cases is 734,000 that is again compatible with the range of values reported in Ref.[6]. While the estimates presented in Refs. [5] and [6] are based on a homogeneous mixing approach within the entire Mexico, the approach used here is a spatially structured model that just in Mexico counts 65 different census areas. These census areas are not equally connected internationally and between them. The number of cases relevant for the international spread of infected individuals are mostly in census areas close to international transportation hubs. Poorly connected regions of Mexico on the other hand, while experiencing a  considerable number of cases, would contribute only marginally to the International spread of cases. This observation readily explains why single population calculations that match the detection of imported cases with the local prevalence are necessarily underestimating the latter quantity.  

While GLEaM takes into account a higher level of geographical organization than previous approaches, its estimates still contain a number of assumptions and approximations. The contagion within each census area is approximated by means of a homogeneous mixing process. Once a person arrives at a census area by plane, he/she comes integrated into the local population. This implies that, as in [6], the travelers and the local population are equally exposed to the disease. Finally, the model considers each individual as independent and the possibility of cluster cases is not considered. Despite these shortcomings and other necessary uncertainties, GLEaM predictions might provide additional information for a better understanding of the early evolution of the present pandemic. Despite the different approximations used here and in Ref.[6], both approaches are providing support to the possibility of a reporting ratio of infected cases in Mexico as low as 1 in 100, in agreement with prior estimates [2]. This finding is important when evaluating the massive amount of data which are now being collected in a large number of countries around the world. We can easily imagine that the reporting rate as well as any estimate of the cumulative attack rate in most of the countries could be easily underestimated by orders of magnitude. 

## Acknowledgments

The authors thank IATA and OAG for providing their databases. We are also grateful to the Staff of the Big Red Computer and the Computational Facilities at Indiana University, as well as to Ciro Cattuto for his support with the computational infrastructure at the ISI Foundation. 

## Funding

This work has been partially supported by the NIH, the NSF, the Lilly Endowment Foundation, DTRA, the ERC project EpiFor and the FET projects Epiwork and Dynanets. The funders had no role in the preparation of the article.

## Competing interests

AV is consulting and has a research agreement with Abbott for the modeling of H1N1 diffusion. The other authors have declared that no competing interests exist.

## Tables

**Table:** 

** Number imported cases (May 8th) **	** USA **	** UK **	** France **	** Germany **	** Brazil**
Simulation Results	0 - 534	0 - 44	0 - 62	0 - 55	0 - 45
Surveillance data	85 (170)	17	11	9	3

    **Table 1. - Cumulative number of imported cases from Mexico shown as the 99% reference range over 2,000 realizations on May 8 for a few countries.** The simulations are obtained with the best estimate parameters of the baseline case of Ref. [9] and R0=1.75 [95%CI 1.64 to 1.88]. The number of imported infected individuals and of independent clusters correspond to the data given in Ref. [6] for US, and UK and the values in [23] for France, in [22] for Germany and in [24] for Brazil. No data was available to assess the possible presence of clusters in Germany and France. In the USA we report in parentheses the revised number considering the rate of unknown travel history in confirmed cases.

**Table:** 

	**Number of symptomatic cases in Mexico (Apr. the 30th)**
Simulation Results	[121,000 - 1,394,000]
Lower bound range of Ref. [6]	113,000-375,000
Estimate of Ref. [5]*	2,000 – 280,000
Mexican official report [7] (confirmed cases)	3,350

**Table 2. Predictions of GLEaM for the size of the epidemic in Mexico on April 30 in thousands of cases and comparison with other approaches and with empirical data.** The simulations are obtained with the best estimate parameters of the baseline case of Ref. [9] and show the 95% reference range over 2,000 stochastic realizations. The results are compared with the lower bound estimate range in [6], the estimate provided in Ref. [5] and the number of confirmed cases given by official reports [27]. *The interval provided for Ref.[5] is obtained by merging the results reported in the paper under different assumptions and including the 95% CI.    
